# Pain of the Loins

**Published:** 1844-09

**Authors:** 

**Affiliations:** Southampton


### PRACTICAL MEDICINE, &c.

*Pain of the Loins.* By Dr. Oke, Southampton.—Perhaps there is no system more commonly met with in practice than pain in the loins, which is usually and at once attributed to bile, gravel, or rheumatism; but as it may be also derived from other causes left out in a hasty decision, I shall enumerate them, and endeavor to point out the symptoms by which each may be distinguished. Pain of the loins may be derived from the muscles, from the liver, from the duodenum, from the kidneys, from the colon, from the uterus, from the aorta, from the spine, or from matter collected on the psoas muscle independent of spinal disease. In order to arrive at its true cause, we must endeavor to ascertain what function is principally involved, which will at once lead us to it.

*If the pain be rheumatic*, it will be increased by pressure, and by the slightest action of the muscles affected. There will probably be also rheumatism in other parts of the body, the system will not evince much disorder, the urine will be high colored, and deposite a lateritious sediment.

*If derived from the hepatic function*, the pain will shoot upwards along the splanchnic nerves to the scapulæ; the alvine evacuations will be either deficient in, or exuberant with, bile; or show a morbid quality of that secretion; the urine will have a bilious tinge; there may be congestion of the hæmorrhoidal veins; and the spirits will be depressed.

*If from the duodenal function*, three or four hours after a meal the pain will be aggravated, shooting through towards the right side of the abdomen, and remaining till the food has passed into the jejunum. Dyspeptic symptoms will prevail, and there will frequently be painful pustules breaking out about the face. I have lately met with a case in which the boils were extremely annoying.

*If from the kidneys*, the pain will shoot down the course of the spermatic nerves towards the round ligament in the female, and towards the testis in the male, which will often be retracted by the action of the spermatic nerves upon the cremaster muscle. There will be more or less irritation communicated to the mucous membrane of the bladder. The urine also will be diagnostic in this instance; it may deposit mucus, calculous matter, blood, pus, or albumen, according to the nature of the case; or it may be otherwise morbid in its constitution.

*If from the uterus*, the pain of the back will arise either from disordered function or disease of that organ. In the former case the pain will be of a neuralgic character, will return in forcing paroxysms extending around the hips and hypogastric region, will be attended with hysteria, and often with increased quantity of the menstrual discharge. In the latter case the pain will be *constant* and severe, extending along the anterior crural nerve half way down the thighs. There will be a thin, offensive discharge from the vagina. The countenance will be wan and sallow, exhibiting the wear and tear of organic lesion.

*If from the colon*, there will be constipation, and inflation in the course of the bowels, or the fœcal discharges will be of small diameter, or there will be soreness of the intestine under pressure, especially at its ascending or descending portions, accompanied by mucus, or shreds of lymph in the from of boiled vermicelli, amongst the excretions.

*If from the arterial dilatation*, an abnormal pulsation of the vessel involved—the aorta, for instance—may possibly be detected by auscultation in the incipient stage of the disease, *if such were suspected;* but in a large majority of cases such a cause may reasonably escape the attention of the ablest surgeon, from there being no tangible symptom that might lead him to suspect it; and even after the dilatation has considerably advanced, it may be sufficiently large to press upon and disturb the spermatic nerves, but not large enough to project and pulsate externally, and this may, at this stage, be confounded with diseases of the renal function. A few years ago I met with a case of this kind in a man of middle age. The pain had been constant and wearing, shooting from the loins down the course of the spermatic nerves, and for a considerable time was reasonably attributed to the renal function, especially as there had been constant disturbance of this function.

At length the aneurismal sac began to approach the surface, and then, of course, the cause became apparent.

*If from the disease of the spinal column*, the pain will be aggravated by percussing the spinous processes at this part of the spine, or by suddenly striking the toes against an uneven surface. There will be involuntary action of the muscles, especially of the flexors of the legs, diminished temperature, abnormal feelings, and more or less loss of power of the lower limbs. Should there be at the same time any unnatural projection of the spinous processes, the disease will be confirmed.

*If from a collection of matter upon the psoas muscle, unconnected with spinal disease*, the pain will be continued, dull, and deep-seated, extending from the loins down the psoæ, or in whatever direction the matter may have taken its course. The pain will be aggravated by flexing the thigh towards the abdomen, and there will be difficulty in walking; moreover, there will be marks of a strumous habit, and more or less symptoms of hectic fever. Should any fluctuating tumor present at the groin, or at any other point where the matter may find its way out of the body, it will be conclusive as to the nature of the case.—*Braithwaite's Med. Retrospect, from Prov. Med. J., Feb.* 17, 1844, *p.* 384.

